# Internalising problems and engagement among preschool children: trajectories of several functional outcomes in a Swedish longitudinal study

**DOI:** 10.1186/s12887-026-06762-1

**Published:** 2026-03-31

**Authors:** Andrea Markkula, Johan Isaksson, Berit M. Gustafsson

**Affiliations:** 1 Department of Child and Adolescent Psychiatry, Jönköping, Region Jönköping County Sweden; 2 Department of Women’s and Children’s Health, Karolinska Institutet & Stockholm Health Care Services, Center of Neurodevelopmental Disorders (KIND), Centre for Psychiatry Research, Stockholm, Region Stockholm Sweden; 3 Department of Medical Sciences, Child and Adolescent Psychiatry, Uppsala University, Uppsala, Sweden; 4 Department of Child and Adolescent Psychiatry and Department of Biomedical and Clinical Sciences , Linköping University, Linköping, Sweden

**Keywords:** Engagement, Preschool, Internalising problems, Developmental trajectories, Child development, Internalising problem, Engagement, Developmental trajectories

## Abstract

**Background:**

Internalising problems and engagement issues are prevalent among preschool children but often go undetected and untreated. There is a notable lack of longitudinal studies examining their long-term impact on functional outcomes and disability. Further knowledge in this area could support the identification of children at risk and inform the development of targeted interventions.

**Aim:**

This study aimed to investigate the longitudinal associations between emotional problems and low engagement on functional outcomes, while controlling for potential confounding variables.

**Method:**

Preschool teachers assessed a representative cohort of 617 Swedish preschool children (317 boys and 300 girls, aged 24–71 months). The study consisted of three assessment waves. At baseline (Wave 1), teachers provided ratings of children’s emotional problems, engagement levels, and functional outcomes. Functional outcomes were then reassessed at Waves 2 and 3. Baseline emotional problems and low engagement were used to define risk groups. Associations between these baseline risk groups and functional outcomes across waves were analysed using Generalised Linear Mixed Models (GLMMs).

**Results:**

In an interaction model, children with both emotional problems and low engagement exhibited the greatest functional outcome improvement over time (b = 0.063; 95% CI [0.038, 0.088] p < 0.001), suggesting potential for developmental catch-up. In a model excluding interaction terms, both emotional problems and low engagement were associated with lower ratings on functional outcomes. Additionally, increasing age, being female, and being a native speaker were associated with higher functional outcomes.

**Supplementary Information:**

The online version contains supplementary material available at 10.1186/s12887-026-06762-1.

## Introduction

Epidemiological studies indicate that 16–18% of children aged 1 to 5 years experience mental health problems, with over half of these cases being severe [1]. Psychopathological issues in infants and toddlers often manifest within the socioemotional domain and tend to persist over time [2, 3]. Children exhibiting early signs of mental health problems are at high risk of developing symptoms in the same or overlapping areas as they grow older [3]. These issues may have long-term implications [3, 4]. Internalising problems encompass a cluster of symptoms reflecting difficulties in regulating emotions and moods, including anxiety, guilt, worry, sadness, and depression [5]. These problems are common among children and adolescents and are associated with various negative outcomes, including behavioural difficulties and child psychopathology [6–9]. Despite this, the understanding of internalising problems in young children has been considered to lag behind compared to other indicators of mental health issues in children [10].

Engagement, often defined as “the amount of time children spend interacting appropriately with their environment at different levels of competence”, is a multidimensional construct encompassing behavioural, emotional, and cognitive components [11, 12]. Emerging evidence suggests that engagement not only plays a crucial role in child development and learning [12], but has also been shown to be interrelated with psychopathology in children [13, 14].

Negative long-term outcomes such as poor academic performance, behavioural difficulties, and later psychopathology have been linked to both internalising problems and low engagement in childhood [6–8, 13, 14]. There is also evidence suggesting that emotional problems and engagement may be interconnected, with each potentially influencing and being influenced by the other [15–17]; however, the exact nature and direction of this relationship remain areas of ongoing research.

The concept of functioning and disability reflects a person’s ability to carry out everyday activities and participate in social life, shaped by dynamic interactions between body function, environment, and personal factors [18]. The International Classification of Functioning, Disability and Health for Children and Youth (ICF-CY) provides a holistic framework that not only includes physiological factors, activities and participation but is also specifically adapted to children’s developmental circumstances [18]. In young children, applying this framework to assess and plan early interventions has been shown to support individualized strategies and improve developmental outcomes [19].

Both internalising problems and engagement issues in young children often go undetected and without interventions [10, 20–23]. Consequently, their impact on functional outcomes and disability remains unclear. In particular, few studies have examined how these early difficulties affect functioning over time using structured frameworks, such as the ICF-CY. There is also a lack of longitudinal studies focusing on preschool-aged children in this context. Further knowledge in this area could aid in assessing the need for targeted interventions for children at risk of long-term negative consequences. Moreover, research indicates that internalising problems and engagement manifest differently across genders, even in early childhood [24, 25]. Girls tend to exhibit higher levels of internalising symptoms such as anxiety and social withdrawal, while boys are more likely to show lower engagement in preschool activities [26–28]. These gender-specific patterns highlight the importance of considering gender when assessing and addressing emotional problems and engagement in young children.

In Sweden, approximately 80–90% of children aged 1–5 years attend preschool. Preschool staff possess a high level of expertise in child development, making the preschool setting an ideal environment for identifying early signs of mental health or behavioural difficulties in young children [22]. This study aimed to investigate the longitudinal associations of emotional problems and low engagement with functional outcomes within a preschool context, while adjusting for potential confounders, including sex, age, waves and native language (Swedish). Specifically, we investigate whether children identified at baseline as having emotional difficulties and/or low engagement differ over time in key functional outcomes compared to their peers. We hypothesise that both emotional difficulties and low engagement will independently relate to poorer functional outcomes longitudinally, and that children exhibiting both risk factors at baseline will show the worst trajectories.

## Method

### Procedure

The data used in this paper were collected as part of the longitudinal research project “Early Detection – Early Intervention in Preschools” (TUTI) [29]. Preschools from a stratified sample of six differently sized Swedish municipalities, representing large, medium and smaller municipalities. The preschool management in each municipality was contacted, and their consent was requested for participation of their preschool units. The preschool teachers then asked all parents for their written informed consent to include their child in the study. Regarding all instruments, teachers were required to have known the child for at least six months, and were asked to base their ratings on at least the two preceding weeks [22]. The children were followed for up to three yearly assessments (baseline, 1-year follow-up, and 2-year follow-up which were defined as wave 1–3) via preschool teachers. There were 311 preschool teachers participating in all waves. The responsible teacher, who knew the child best, was selected as the informant teacher in the study. On average, each teacher reported on two children.

### Participants

In the first data collection set, parents of 1615 children were invited to participate. The parents of 845 of these children (52%) subsequently gave their consent to include their child in the study i.e. the preschool teachers completed questionnaires about the child’s functioning and behaviours during preschool years. In this study, teachers completed the Strengths and Difficulties Questionnaire (SDQ) emotional subscale, the Children’s Engagement Questionnaire (CEQ), and functional outcomes based on the International Classification of Functioning Disability and Health – Children and Youth Version (ICF‑CY) at Wave 1. At Waves 2 and 3, only functional outcomes were collected. We excluded children younger than 2 years (*n* = 129). Of the remaining 716 children, 13 lacked data on native language, 32 for CEQ, 58 for SDQ, and for ICF-CY 2 lacked data on wave 1, 316 for wave 2, and 448 for wave 3. Little’s MCAR test was significant (χ2 = 308.26, *p* < 0.001), which indicates that data was not missing completely at random. Those missing all data for the independent and the dependent variables needed for the Generalized Linear Mixed Models (GLMMs), and thus excluded from the main analyses (*n* = 99), were more likely to be non-native speakers (χ2 = 42.43, *p* < 0.001). An attrition analysis was conducted including the variables parent-rated child distress, preschool staff-rated collaboration with parents, and the presence of additional support staff in the preschool setting. Parents of children with incomplete data rated their child’s experienced distress as significantly lower (M = 8.03, SD = 1.93, df = 566) than parents of children with complete data (M = 8.61, SD = 2.06; t = 2.11, *p* = 0.035). Additionally, preschool staff rated the level of collaboration between the preschool and the parents for those children with incomplete data as significantly lower (M = 16.38, SD = 3.02 vs. M = 17.50, SD = 2.55; t = 3.25, *p* = 0.002, df = 684). It was also more common for preschools caring for children with missing data to have additional support staff present (44.1% vs. 25.0%; χ² = 14.66, *p* < 0.001, df = 1). No differences in the other study variables (sex, age, emotional problems, low engagement or functional outcomes) were found. Hence, the final sample constituted of 317 boys and 300 girls with a mean age of 45.6 months (SD = 13.45, age range 24–71). In this sample of 617 children, all had data on ICF-CY for wave 1, 355 for wave 2 and 237 for wave 3. The reason for non-response at follow-up varies, i.e. children are no longer preschool children, children change preschools, or preschool teachers are unable to complete the questionnaires due to their work situation at wave 2 and/or wave 3.

At the time of investigation, a total of 90.3% of the children lived with both their biological parents, 3.8% lived alternately with each parent, 0.2% lived with only their father, 4.3% lived with only their mother, and 0.7% lived with someone else. Altogether, 24.3% of the children had a mother tongue other than Swedish and, according to preschool teachers’ estimation, 4.2% received special support in preschool. These figures correspond with Statistics Sweden (SCB) (Statistics Sweden, 2013; Swedish National Agency for Education, 2017) estimates for this age group.

In the complete cohort, the preschool group mean size was 22 (range 8–50) children, with a mean of 4 preschool teachers (range 1–7) in the group. The child-to-teacher ratio averaged 5.6 children/teacher. During the time that the study was conducted, 49 preschools participated, with 110 preschool classes. Approximately 47% of the children lived in small municipalities (< 50,000), 45% in medium-sized municipalities (50,000–200,000) and 8% in large municipalities (> 200,000). In Sweden as a whole, 43% live in small municipalities, 16% in medium ones and 41% in large; thus, large municipalities are under-represented and medium-sized ones are over-represented in our sample.

### Instruments

#### Strengths and Difficulties Questionnaire (SDQ)

The Strengths and Difficulties Questionnaire (SDQ) is widely used for screening emotional and behavioural difficulties in children and young people. It consists of 25 items that are rated on a 3-point scale by parents, teachers, or by the child themselves (from 11 years of age). Each item describes a specific behaviour or emotional symptom, and the rater indicates how well the statement applies to the child by selecting “Not true” (0), “Somewhat true” (1), or “Certainly true” (2). Scores from related items are then summed to produce subscale scores, such as those for emotional problems or conduct difficulties, which help identify areas where the child may be experiencing challenges.

The SDQ for ages 3–18 years displays good construct and concurrent validity, as well as some evidence of predictive validity [30–33]. In the present study, the emotional subscale of the SDQ teacher version for children aged 2–4 [34] was administered at baseline (wave 1). The SDQ emotional subscale is made up of five items relating to depressive symptoms, worry, fear, nervousness, and somatic symptoms, it has been shown to appropriately measure the risk of emotional disorders in children [33, 35]. It has been shown to assess internalising problems in a satisfactory way [36].

The SDQ has been translated into Swedish and validated for parental and teacher use for children between 1 and 17 years of age; it has demonstrated good psychometric properties [29, 37–40]. The SDQ for parents has been used in a normative sample of children aged 2–5 years in Sweden, and here risk groups are reported as percentiles to identify them [41]. The SDQ has been confirmed as having satisfactory psychometric properties in identifying 3- and 4-year-olds with emotional difficulties [33].

In accordance with SDQ guidelines, a value of 3 and above was considered at risk regarding emotional problems for children aged 2–4 years, whereas a cutoff of 4 and above was set for children older than 4 years [34]. Cronbach Alpha for the emotional subscale was 0.60.

#### The Children’s Engagement Questionnaire (CEQ)

Preschool teachers used the Child Engagement Questionnaire (CEQ) [42] in order to measure global child engagement. Designed in the USA, the instrument was used to rate how children typically spend their time when in preschool, assessing a child’s levels of engagement with peers, adults, and materials. Behavioural examples are included for each item to clarify the intent of the assessment. Previous research on the CEQ has identified four key factors underlying the assessment: Competence, exemplified by items such as “Attempts to complete tasks, even if they require considerable time and effort”; Persistence, as demonstrated by items like “Attempts to engage an adult in activities”; Undifferentiated Behaviour, illustrated by items such as " Plays with objects in a simple manner”; and Attention, evidenced by items like “Observes or listens attentively to other children” [43].

In the present study, teachers completed the CEQ at baseline (wave 1). Minor adaptations were employed in the translation of CEQ into Swedish, which resulted in the use of 29 of the original 32 items [22]. The response alternatives for the Swedish four-point Likert scale are (1) almost never happens (2) sometimes happens (3) happens quite often or (4) happens very often. Total engagement score corresponds to the sum of the item scores. The score could range between 1 and 116 where a lower score indicate lower engagement. Earlier studies have reported good content and construct validity, as well as intra-rate reliability, for the Swedish translation of CEQ [22, 44, 45]. Cronbach Alpha for the engagement scale was 0.77. Children who scored in the lower quintile of engagement ratings in the CEQ-questionnaire were considered at risk regarding engagement. This dichotomization of the scale based on quintiles is in line with the scoring distribution of the SDQ, where 20% of the population is categorized as slightly low to very low.

#### The International Classification of Functioning, Disability and Health: Children and Youth Version (ICF-CY)

The World Health Organization (WHO) International Classification of Functioning, Disability and Health (ICF) was developed to serve as a comprehensive framework for the components of functioning and disability for all health-related conditions [18, 46]. Derived from the ICF, the ICF version for Children and Youth (ICF-CY) was later developed to capture the particular situation of the developing child by adding categories and expanding on descriptions [18, 46]. ICF-CY serves as tool to understand health and functioning while still addressing age appropriate developmental aspects of children’s abilities in various life situations [18].

The ICF-CY consists of 1685 categories. As the extensive scope of the complete ICF-CY constrains its applicability, the implementation strategy has been suggested to be that the use of ICF must be customized to meet the specific requirements of different user groups [18, 46]. Tailored ICF-CY code sets have been developed provide a standardized marker of childhood disability across various developmental stages and context [47]. Using these ICF-CY Code Set, this study used seven preschool teacher items rated at waves 1–3 to assess developmental delay regarding bodily function (“The child uses their arms and hands”, ”The child moves in different ways”, ”The child responds to pain”), cognition (“The child understands basic concepts”, “The child can initiate, perform, and complete a task”, ”The child manages transitions between tasks”), and language (“The child can converse”) [47]. These were responded to on a scale of: (1) “not true at all” (2) “partly true” and (3) “completely true”, with a possible range between 1 and 21 for the total score, and a lower score indicating lower child functioning. Due to the conceptual diversity of the seven ICF-CY items, strict unidimensionality cannot be assumed. Hence, complementary domain-specific models were included. Primary analyses were conducted using the total score to capture global functioning as an outcome, whereas complementary models were performed using the separate subdomains (bodily functions, cognition, and language) to provide a more nuanced understanding of domain-specific trajectories. Cronbach Alpha for the scale was 0.80 at all waves, indicating a good internal reliability. Further, a confirmation factor analysis based on the 7 items at baseline, using Diagonally Weighted Least Squares (DWLS) as estimator yield a RMSEA of 0.079, a CFI of 0.995, and a TLI of 0.995 (χ2 = 81.52 (df = 14), *p* < 0.001). These values indicate that a one-factor model provides an acceptable fit according to RMSEA and an excellent fit according to CFI and TLI. While this suggests that the items are reasonably coherent, the significant chi-square test and the conceptual diversity of the items (covering bodily function, cognition, and language) mean that strict unidimensionality cannot be assumed.

### Data analysis plan

Analyses were performed using SPSS v. 29 and two-tailed tests with p values < 0.05 considered to be significant. Comparisons between children with and without emotional problems or low engagement were made regarding age, sex, family situation, and native language status. Independent-samples t-tests were used for continuous variables, and Pearson’s chi-square tests were applied for categorical (binary) variables. Spearman’s rho was used for assessing correlations between the study variables.

The outcome variables, functional outcomes, were not normally distributed (Kolmogorov–Smirnov test: D = 0.196–0.264, *p* < 0.001) and exhibited a left-skewed distribution (skewness ratio = −7.74 to −14.07). To assess the association between emotional problems (coded as 1 if above established cutoff) and low engagement (coded as 1 if in the lower quintile of engagement ratings) at baseline with functional outcomes across three waves (i.e., baseline, 1-year follow-up, and 2-year follow-up), Generalized Linear Mixed Models (GLMMs) were employed. Since GLMMs use maximum likelihood estimation—which allows the inclusion of all available data for each subject, even if some time points or variables are missing—and account for random effects, we did not impute missing data but instead used the available (i.e., incomplete) data. A gamma distribution with a log link was used, as this model demonstrated the best goodness-of-fit based on Akaike information criterion and by visually inspecting the residuals with histograms and Q-Q plots. Covariates included in the GLMM were child age, sex (male or female), native language status (native or non-native), and waves as a continuous variable. In line with recommendations for hierarchical testing of interaction models, we first examined a three-way interaction (time × emotional problems × low engagement) while adjusting for covariates, and lower-order interactions (time × emotional problems, time × low engagement, and emotional problems × low engagement) and main effects were considered conditional on this effect. In addition, we re-calculated the GLMMs as a complementary analysis in order to present main effects from model without interactions. As a sensitivity analysis, the GLMM was recalculated using the subscales of functional outcomes—namely, bodily problems, cognitive issues, and languange problems.

### Ethical considerations

The study was approved by the Regional Ethical Review Board in Linköping (Dnr 2012/199–31). Written informed consent was obtained from preschool management, teachers, and both parents of each child. Questionnaires were coded, and the key was stored separately. Teachers were instructed to refer children with newly identified mental health concerns to child healthcare. All procedures complied with national ethical standards and the Helsinki Declaration, as revised in 2008.

## Results

The total amount of data points varied depending on wave and questionnaires. Not all participants answered the full battery. All descriptive analyses were made from the samples available data. The GLMM utilizes the datapoints post data manipulation (*n* = 1209).

### Group characteristics

Child characteristics depending on emotional problems and engagement status are presented in Table 1 and correlations between the study variables are presented in Supplementary Table 1. In total, 12.2% of the sample were classified as having emotional problems according to the SDQ cut-offs, children in the lower quintile of CEQ scores were coded as having low engagement. In total, 446 were classified as not having emotional problems or low engagement, 96 as not having emotional problems but low engagement, 48 as having emotional problems but not low engagement, and 27 as having both emotional problems and low engagement. Children classified as having emotional problems were significantly younger than those without emotional problems (t = 4.86, *p* < 0.001). Similarly, children with a low engagement were significantly younger than their more engaged peers (t = 5.82, *p* < 0.001). Additionally, children with low engagement were more often boys (χ² = 5.67; *p* = 0.017). There were no differences in family situation (i.e., living with both parents or not) between the groups; however, participants with a low engagement were less often native speakers (χ² = 6.80; *p* = 0.009).

**Table 1:** Child characteristics by emotional problems and engagement status

	All,*n* = 617	Emotional problems,*n* = 75	Noemotional problems,*n* = 542	Low engagement,*n* = 123	No low engagement,*n* = 494
Sex (female)	300 (48.6%)	40 (53.3%)	260 (48.0%)	48 (39.0%)	252 (51.0%)
Age in months, mean (SD)	45.6 (13.4)	39.8 (10.6)	46.4 (13.6)	39.5 (12.8)	47.1 (13.2)
Native language, Swedish	467 (75.7%)	58 (77.3)	409 (75.5%)	82 (66.7%)	385 (77.9%)
Living with both parents^d^	557 (91.2%)	70 (93.3%)	487 (90.9%)	110 (90.2%)	447 (91.4%)
Functioning wave 1, mean (SD)^a^	19.15 (2.30)	17.89 (2.70)	19.32 (2.19)	16.49 (2.50)	19.81 (1.70)
Functioning wave 2, mean (SD)^b^	19.74 (1.99)	19.50 (1.80)	19.77 (2.02)	18.14 (2.74)	20.14 (1.51)
Functioning wave 3, mean (SD)^c^	19.55 (2.14)	19.15 (2.41)	19.62 (2.09)	18.53 (2.74)	20.01 (1.62)

Emotional problems (SDQ) and Engagement (CEQ) were collected at baseline (wave 1). ICF‑CY functional outcomes were collected at waves 1–3

^a^ *n* = 617, ^b^ *n* = 355, ^c^ *n* = 237, ^d^ *n* = 611

### Primary outcomes

Estimated means for functional outcomes by both emotional problems and low engagement from the full model including covariates and interaction effects are presented in Supplementary Table 2. The primary analysis is presented in Table 2 and revealed a significant three-way interaction between time, emotional problems and low engagement on functional outcomes. This interaction indicated that children with both emotional problems and lower engagement, showed a greater increase in functioning over the three years compared to children with higher engagement and no emotional problems at baseline. Given the presence of this higher-order interaction, main effects and lower-order interactions must be interpreted as conditional to this higher-order interaction. Older children, girls, and native speakers had higher functional outcome ratings on average compared to younger children, boys, and non-native speakers. As a complementary analysis, we also examined a model without interaction terms (Supplementary Table 3). In this model, both emotional problems and low engagement were associated with lower ratings on functional outcomes, providing additional information about the overall main effects when considered independently of interactions.

**Table 2:** Results from Generalized Linear Mixed Models with emotional problems and low engagement at baseline, covariates, interactions, and functional outcomes

	Functional outcomes	Bodily function	Cognition	Language (verbal)
	b, 95% CI	b, 95% CI	b, 95% CI	b, 95% CI
Intercept	2.855; 2.821, 2.889	2.148; 2.127, 2.168	1.917; 1.862, 1.971	0.636; 0.548, 0.723
Emotional problems	− 0.027; − 0.069, 0.016	− 0.010; − 0.038, 0.019	− 0.051; − 0.120, 0.018	− 0.005; − 0.121, 0.112
Low engagement	− 0.196; − 0.227, − 0.165***	− 0.059; − 0.080, − 0.038***	− 0.276; − 0.326, − 0.226***	− 0.532; − 0.616, − 0.447***
Sex (female)	0.017; 0.002, 0.031*	0.000; − 0.008, 0.008	0.029; 0.005, 0.052*	0.033; − 0.069, 0.003
Age	0.003; 0.002, 0.003***	0.001; 0.000, 0.001***	0.004; 0.003, 0.005***	0.007; 0.006, 0.009***
Native language, Swedish	0.029; 0.013, 0.046***	0.008; − 0.001, 0.018	0.057; 0.030, 0.084***	0.100; 0.058, 0.142***
Waves	0.020; 0.013, 0.027***	0.007; 0.001, 0.012*	0.026; 0.015, 0.038***	0.050; 0.031, 0.070***
Emotional problem x low engagement	− 0.046; − 0.119, 0.026	− 0.028; − 0.077, 0.021	− 0.076; − 0.193, 0.041	− 0.080; − 0.278, 0.117
Waves x low engagement x Emotional problems	0.063; 0.038, 0.088***	0.019; 0.001, 0.037*	0.102; 0.062, 0.142***	0.166; 0.097, 0.236***
Waves x low engagement x no Emotional problems	0.043; 0.030, 0.057***	0.012; 0.002, 0.022*	0.059; 0.037, 0.081***	0.130; 0.091, 0.169***
Waves x no low engagement x Emotional problems	0.005; − 0.016, 0.025	0.001; − 0.013, 0.016	0.010; − 0.022, 0.043	− 0.009; − 0.067, 0.049

The lower ordered interactions of time × emotional problems and time x low engagement are included in the model but not separately estimable. *p < 0.05; ***p < 0.001. The GLMM utilizes the datapoints post data manipulation (*n* = 1209). In total, 617 children were included, where all had data on ICF-CY for wave 1, 355 for wave 2 and 237 for wave 3. Emotional problems (SDQ) and Engagement (CEQ) were collected at baseline (wave 1). ICF‑CY functional outcomes were collected at waves 1–3

### Sensitivity analyses

**Fig. 1:**
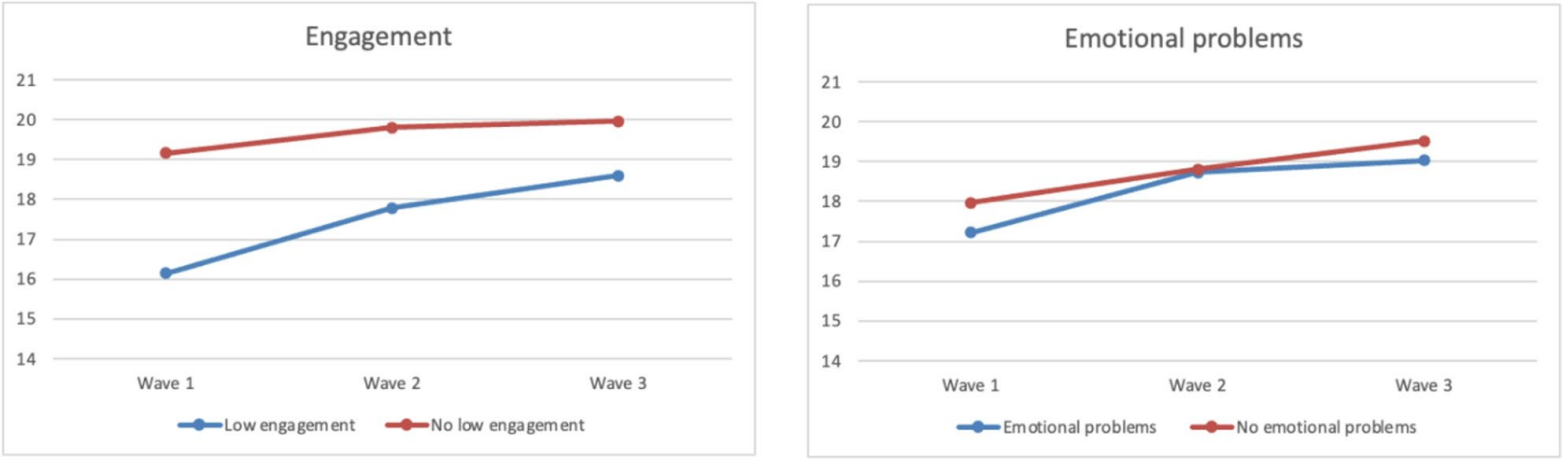
Graphical representation of the two-way interaction effects for functional outcomes by both emotional problems and low engagement

Figure 1 depicts the the two-way interaction analysis of time and emotional problems as well as time and low engagement. Table 2 presents the results of the GLMMs for the different functional outcome domains. Consistent with the primary analysis, a significant interaction between time, emotional problems and low engagement was observed across all functional areas, indicating that children with lower engagement, and both emotional problems and lower engagement, showed a greater increase in functioning over the three years compared to children with no emotional problems and higher engagement at baseline. This finding indicates that the effects of emotional problems and engagement on functional outcomes were conditional on each other and on time. Additionally, increasing age and time were positively associated with all functional outcomes. Being female was also linked to better ratings in cognition, while being native speaking was also linked to better ratings in cognition and language function. When excluding interactions as a complementary analysis, emotional problems at baseline was associated with lower ratings in the domains of body function and cognition, and low engagement with lower ratings across all functional areas (Supplementary Table 3).

## Discussion

This longitudinal study explored the associations between emotional problems and low engagement in preschool children, and their influence on functional outcomes across three waves. Interaction effects were found where children with both emotional problems and low engagement exhibited the greatest improvement in functional outcomes over time, suggesting potential for developmental catch-up. Low engagement at baseline was associated with lower functional outcomes, while increasing age, being female, and being a native speaker were associated with higher functional outcome ratings in the model. These findings emphasize the unique and potentially modifiable role of early engagement in shaping children’s developmental trajectories, and partially support previous research on the long-term consequences of early behavioral challenges [6–8, 13, 14].

In models that account for interactions between emotional problems, engagement, and time, the findings indicate that the effects of emotional problems and engagement on functional outcomes are interdependent and improve over time. Specifically, the three-way interaction showed that children with lower engagement and children having both emotional problems and lower engagement had a greater increase in functioning over the three years compared to those with no emotional problems and higher engagement. This finding goes against our initial hypothesis stating that children exhibiting both risk factors at baseline will show the worst trajectories and suggests that the strength of the associations between emotional problems, low engagement, and functional outcomes may diminish over time. This pattern could partly reflect regression to the mean, especially given that the risk groups were defined based on extreme baseline scores. However, several factors support the interpretation that these are meaningful longitudinal associations rather than statistical artifacts. The use of GLMMs, which account for individual variability and include all available data points, helps mitigate regression to the mean. Additionally, the inclusion of relevant covariates and the persistence of group differences across multiple waves and functional domains further strengthen the validity of the findings.

Encouragingly, this pattern may also reflect meaningful developmental progress. One plausible explanation is that informal or non-systematic interventions already exist within Swedish preschools [18, 27]. Given the high level of teacher training and the structured, inclusive nature of preschool environments, children with early difficulties may receive increased attention, support, and opportunities for engagement and skill development—even in the absence of formalized intervention programs. These findings underscore the potential of everyday preschool practices to buffer against early vulnerabilities and promote developmental catch-up. Incorporating systematic screening tools like the SDQ and CEQ into preschool routines could help even more in identifying children at risk and initiating timely interventions. Nevertheless, it is important to note that this interpretation remains hypothetical, and future research should examine whether characteristics of preschool environment indeed contribute to the improvement observed in this subgroup.

When interaction effects were not considered, especially low engagement, but also emotional problems, were associated with lower ratings in the functional domains of body function and cognition. Low engagement at baseline was consistently associated with lower functioning across all domains—suggesting that engagement is a meaningful indicator of developmental outcomes in early childhood. These findings support theoretical models that view engagement as a multidimensional construct that underpins learning, behaviour, and emotional regulation [48]. The mechanisms underlying the association between low engagement and poorer functional outcomes may involve reduced opportunities for active learning, social interaction, and skill development. Interestingly, although lower engagement was associated with lower functioning, low engaged children showed more pronounced gains over time compared to their highly engaged peers. This may reflect the capacity for developmental catch-up, possibly facilitated by increased adult attention, targeted support, or maturation [12, 49]. These findings echo research suggesting that early engagement difficulties, while concerning, are amenable to intervention and may be mitigated through structured preschool environments [48]. As for emotional problems, findings from the model without interactions are consistent with previous research showing that internalizing symptoms, such as anxiety, can negatively impact functional outcomes in children [50, 51]. Overall, the findings highlight that the relationships between these variables and functional outcomes may be more complex than they may initially appear. Examining each variable in isolation risks oversimplifying the dynamics at play and may lead to misleading conclusions about their true associations with functioning.

The analysis revealed that age, being female, and native language status were positively associated with functional outcomes. These results are in line with evidence from several studies that suggests that girls may receive more emotionally rich and linguistically supportive interactions from parents during early childhood, which could contribute to earlier development in socioemotional and language domains compared to boys [49]. Similarly, native speakers may have an advantage in preschool environments where the instructional language matches their home language, thereby facilitating engagement and cognitive processing [45]. This highlights the importance of culturally and linguistically sensitive approaches in early childhood education and intervention planning.

A key strength of this study is its large, representative [29], longitudinal sample drawn from diverse municipalities, along with the use of validated instruments such as the SDQ, CEQ, and ICF-CY [18, 22, 29, 44, 45]. This provided a well-rounded assessment of the children and their development over time. The study also employed robust statistical methods that accounted for missing data and controlled for key demographic variables.

However, several limitations must be acknowledged. The proportion of parents consenting to include their child in the study was only 52%. This was partly due to a stringent inclusion criterion requiring informed consent from both caregivers, a measure taken for ethical reasons. Previous drop-out analyses revealed that children excluded from the study were more likely to have a mother tongue other than Swedish [29]. As a result, the included and excluded groups differ somewhat in composition and may also differ in symptomatology. This selective inclusion may limit the generalizability of the findings. A further limitation relates to the measurement of emotional problems using the SDQ. Although widely used, the emotional subscale includes only five items, which may limit its ability to capture the full range of emotional difficulties in young children. In this study, internal consistency was modest (Cronbach’s alpha = 0.60), suggesting reduced reliability. Moreover, despite the good model fit indicated by the confirmatory factor analysis of the ICF-CY items, the seven items measure diverse domains of functioning and as such internal consistency indices and confirmatory factor analysis may not be the most appropriate validation approaches as these results could be regarded as evidence of coherence rather than definitive proof of unidimensionality. Alternative approaches to assessing functioning, such as the use of questionnaires such as the Adaptive Behavior Assessment System or the Vineland Adaptive Behavior scale, targeting a wide range of functional behaviors across different domains, could provide additional insights into the studied interactions. Further, the dichotomization of continuous SDQ and CEQ scores inevitably reduces variability compared to analyses using continuous measures. This decision was made to enhance clinical interpretability and facilitate risk-group identification. Nevertheless, dichotomization may increase the risk of power loss, particularly in analyses involving interaction terms which may be an issue considering missing data from participants not competing all rating and not participating in all waves. Also, all data were based solely on teacher ratings. While teachers offer valuable insights into children’s behaviour in preschool settings, relying on a single informant may introduce bias. The absence of parent or child reports limits the ability to validate findings across contexts. Future studies should consider multi-informant approaches to enhance validity.

Moreover, we had missing data from participants not competing all rating and not participating in all waves. A high rate of attrition is common in longitudinal studies where individuals are lost during follow-up. In our study, children who were non-native speakers had a higher degree of incomplete data. Although, those that drop-out often differ on several characteristics from those retained [52], which may result in a selection bias, loss to follow-up rarely affects estimates of any found association [52, 53]. Since GLMMs use maximum likelihood estimation—which allows the inclusion of all available data for each subject, even if some time points or variables are missing—and account for random effects, and since methods for adjusting loss of cases, such as multiple imputation, involve assumptions that the data is missing at random (MAR), we made a decision to not impute missing data but instead used the available (i.e., incomplete) data. The MAR assumption may be challenged in our study, as our attrition analysis indicated that missingness was associated with variables such as parent-rated child distress, staff-rated collaboration with parents, and the presence of additional support staff. These variables were however not included in the GLMM due to missing data for several participants, which would have reduced the sample size. Furthermore, other relevant predictors of attrition—such as parental education, socioeconomic status, and family structure—were not available in our dataset. Their omission may limit the robustness of the MAR assumption and introduce bias if these unmeasured factors influence both attrition and outcomes. Future studies should aim to collect more complete data on these and other relevant variables.

Additionally, the follow-up period for this cohort was limited to three years. Studies investigating the trajectory of these functional outcomes beyond this timeframe, especially based on risk group status, would be valuable. Finally, our dataset does not include identifiers for specific teachers or preschool classes, which limits our ability to model potential clustering at the class or teacher level. As a result, clustering effects—such as shared informant bias or within-class dependencies—could not be formally accounted for in our analyses. Future studies with access to more detailed class- or teacher-level identifiers could more thoroughly assess the risk of such dependencies, for example by estimating intraclass correlations or applying three-level models.

Further research should explore the mechanisms through which emotional difficulties and engagement influence developmental outcomes. This includes examining the role of preschool interventions, home environments, and individual child characteristics. Longitudinal follow-up into the school years could provide valuable insights into how early functioning translates into later academic and mental health outcomes.

## Conclusion

This study contributes to the expanding body of research highlighting the critical role of early socioemotional functioning and behavioral engagement in shaping children’s developmental trajectories. The findings indicate that the effects of emotional problems and engagement on functional outcomes in preschool-aged children are interdependent and fluctuate over time. This underscores the importance of early identification and intervention within preschool settings.

Notably, the observed attenuation of associations over time may suggest that some early support mechanisms are already in place. In addition, increasing age was independently associated with improved functional outcomes, indicating that maturation itself may play a beneficial role in children’s development. This highlights the importance of considering developmental timing when designing interventions.

Nevertheless, proactively addressing these challenges during early childhood holds significant potential to positively influence long-term developmental outcomes and reduce the risk of disability and dysfunction in later childhood and adolescence.

## Supplementary Information

Supplementary Material 1.

## Data Availability

The data that support the findings of this study are available upon request from the corresponding author, AM.
