# Supplementary material for: Internalising problems and engagement among preschool children: trajectories of several functional outcomes in a Swedish longitudinal study
Source: BMC Pediatr. 2026 Mar 31;26:287. doi: 10.1186/s12887-026-06762-1 (PMC13063683; doi:10.1186/s12887-026-06762-1)
Supplement: Supplementary file 1 — Supplementary Material 1. [file 12887_2026_6762_MOESM1_ESM.docx]

Supplementary Table 1. Correlations (Spearman’s rho) between the study variables

|  | Gender (female) | Age | Swedish language | Emotional problems | Low engagement | Functioning wave1 | Functioning wave2 |
| --- | --- | --- | --- | --- | --- | --- | --- |
| Age | .100* |  |  |  |  |  |  |
| Swedish language | .022 | .119** |  |  |  |  |  |
| Emotional problems | .035 | -.159*** | .014 |  |  |  |  |
| Low engagement | -.096* | -.233*** | -.105** | .150*** |  |  |  |
| Functioning wave1 | .128** | .464*** | .220*** | -.207*** | -.539*** |  |  |
| Functioning wave2 | .200*** | .364*** | .210*** | -.109* | -.349*** | .641*** |  |
| Functioning wave3 | .154* | .290*** | .189** | -.078 | -.277*** | .495*** | .577*** |

Note n = 617 at wave 1, n = 355 at wave 2, and n = 237 at wave 3.

Supplementary Table 2. Estimated Means (EEM) in the full model for functional outcomes by both emotional problems and low engagement

|  | Wave 1 | Wave 2 | Wave 3 |
| --- | --- | --- | --- |
|  | EEM (standard error) | EEM (standard error) | EEM (standard error) |
| Low engagement | 16.15 (0.18) | 17.78 (0.25) | 18.60 (0.26) |
| No low engagement | 19.16 (0.16) | 19.80 (0.19) | 19.96 (0.22) |
| Emotional problems | 17.22 (0.21) | 18.73 (0.29) | 19.03 (0.31) |
| No emotional problems | 17.97 (0.11) | 18.80 (0.14) | 19.51 (0.15) |

*Note* Time was treated as a continuous variable in the model. For presentation purposes, EEM were computed at the three waves to illustrate group differences over time.

Supplementary Table 3. Results from Generalized Linear Mixed Models with emotional problems and low engagement at baseline, covariates, and functional outcomes.

|  | Functional outcomes | Bodily function | Cognition | Language (verbal) |
| --- | --- | --- | --- | --- |
|  | b, 95% CI | b, 95% CI | b, 95% CI | b, 95% CI |
| Emotional problems | -.026; -.048, -.004* | -.014; -.027, -.002* | -.042; -.077, -.006* | -.021; -.077, .034 |
| Low engagement | -.126; -.144, -.107*** | -.041 -.052, -.031*** | -.178 -.208, -.149*** | -.305 -.351, -.259*** |
| Sex (female) | .018; .003, .032* | .000; -.008, .008 | .030; .006, .053* | .035; -.001, .071 |
| Age | .003; .002, .003*** | .001; .000, .001*** | .004; .003, .005*** | .007; .006, .009*** |
| Native language, Swedish | .031; .014, .048*** | .008; .006, .014*** | .059; .032, .086*** | .102; .060, .144*** |
| Waves | .033; .027, .039*** | .010; .008, .016*** | .046; .037, .055*** | .087; .070, .104*** |
|  |  |  |  |  |

Note. *p < 0.05; ***p < 0.001;
